# The Mysterious Island: Insula and Its Dual Function in Sleep and Wakefulness

**DOI:** 10.3389/fnsys.2020.592660

**Published:** 2021-02-11

**Authors:** Ekaterina V. Levichkina, Irina I. Busygina, Marina L. Pigareva, Ivan N. Pigarev

**Affiliations:** ^1^Institute for Information Transmission Problems (Kharkevich Institute), Russian Academy of Sciences, Moscow, Russia; ^2^Department of Optometry and Vision Sciences, The University of Melbourne, Parkville, VIC, Australia; ^3^Pavlov Institute of Physiology, Russian Academy of Sciences, Saint Petersburg, Russia; ^4^Institute of Higher Nervous Activity and Neurophysiology, Russian Academy of Sciences, Moscow, Russia

**Keywords:** insular cortex, wakefulness, sleep, visceral regulation, visceral theory of sleep, gut-brain communication, interoception

## Abstract

In the recent sleep studies, it was shown that afferentation of many cortical areas switches during sleep to the interoceptive one. However, it was unclear whether the insular cortex, which is often considered as the main cortical visceral representation, maintains the same effective connectivity in both states of vigilance, or processes interoceptive information predominantly in one state. We investigated neuronal responses of the cat insular cortex to electrical stimulations of the intestinal wall delivered during wakefulness and natural sleep. Marked increase was observed in the number of insular neurons responding to this stimulation in sleep comparing to wakefulness, and enlarged amplitudes of evoked local field potentials were found as well. Moreover, most of the cells responding to intestinal stimulation in wakefulness never responded to identical stimuli during sleep and vice versa. It was also shown that applied low intensity intestinal stimulations had never compromised sleep quality. In addition, experiments with microstimulation of the insular cortex and recording of intestinal myoelectric activity demonstrated that effective insula-to-gut propagation also happened only during sleep. On the other hand, the same insular stimulations in wakefulness led to contractions of orofacial muscles. The evoked face movements gradually disappeared in the course of sleep development. These findings demonstrate that pattern of efficient afferent and efferent connections of the insular cortex changes with transition from wakefulness to sleep.

## Introduction

Insular cortex is often considered as the main representation of the visceral systems at the cortical level (Saper, 2002; Aleksandrov and Fedorova, 2003; Bagaev and Aleksandrov, 2006; Craig, 2009; Nieuwenhuys, 2012), following a common approach to classification of the cortical areas according to the dominant input from a particular sensory modality, e.g., visual, auditory, visceral, etc.

However, the current map of the cortical functions is dramatically different from the one widely accepted by the middle of the twentieth century, when proportionally more of the physiological studies were devoted to the investigation of various visceral systems and their cortical representations. In these early experiments, performed in acute conditions under general anesthesia, responses to stimulation of internal organs and visceral nerves were found in frontal, parietal, and temporal cortices (Amassian, 1951; Chernigovskii and Musiashchikova, 1965; historic overview of the frontal cortex involvement in visceral control is presented in the review by Neafsey, 1990). These responses were comparable to the evoked responses to exterosensory stimulation of other modalities in the corresponding primary sensory areas. Visceral responses of the longer latencies—secondary visceral responses—were recorded virtually in all cortical regions. This view at that time was not considered surprising, given a huge number of interoreceptors distributed in various visceral organs (e.g., Chernigovskii, 1960, 1967).

The picture began to change when new electrophysiological techniques, which allowed working with behaving animals without anesthesia, started to replace previous methods. Using these new methods investigators could not find responses to visceral stimuli in the most of these cortical fields. In the previous studies, it was also often noted that better responses to visceral stimuli were recorded at deeper level of anesthesia.

Given these new results, wide cortical representations of the visceral systems were recognized as artifacts of anesthesia, despite that anesthesia usually leads to the opposite effect of disappearance of the responses existing in wakefulness. Nevertheless, the former cortical visceral areas were reattributed as the associative visual, auditory and somatosensory cortical areas, and, in prefrontal cortex, into the areas participating in emotion regulation rather than visceral control. Visceral regulation was largely attributed to hypothalamus, which, despite having multiple connections with cortical areas, was not supposed to bother cortex with visceral matters (e.g., Neafsey, 1990).

At that time, the “last island” where responses to particular visceral stimuli could be recorded in chronic physiological experiments during active wakefulness was the insular cortex (e.g., Bagaev and Aleksandrov, 2006). Since then insular cortex was considered as the main cortical representation of at least cardio-vascular (Ruggiero et al., 1987), gastro-intestinal (Cechetto and Saper, 1987; Aleksandrov et al., 1996; Busygina et al., 2009) and respiratory visceral systems (Aleksandrov and Aleksandrova, 1998; Banzett et al., 2000). Insula has been also recognized as an important part of so called Central Autonomic Network involved in regulation of multiple visceral responses (Benarroch, 1993; Nagai et al., 2010).

However, recent sleep studies cast doubt on the consistency of this conclusion. It was shown that neurons in sensory cortical areas (visual, somatosensory) without any anesthesia switch to processing of the visceral information, but do that only during sleep (Pigarev, 1994; Pigarev et al., 2013). These results let us propose that during sleep propagation of the interoceptive signals opens to all cortical areas, and all cerebral cortex becomes visceral (Pigarev et al., 1997; Pigarev and Pigareva, 2014, 2017). Correspondingly, active efferent cortical projections also should change destination during transition from wakefulness to sleep. From this point of view, connections of multiple cortical areas with visceral organs observed in the mentioned earlier acute experiments, as well as responses of these visceral organs to cortical stimulation, were a physiological reality, and applied anesthesia just “modeled” natural state of sleep.

In this context, position of the insular cortex becomes ambiguous. One option is that insular cortex is an exceptional cortical area keeping constant visceral afferent and efferent connectivity both in wakefulness and in sleep.

However, an alternative and, in our opinion, more probable option is also possible. Afferent and efferent connections of the insular cortex, as in many other investigated cortical areas, may change during transition from one state to another. It is easy to imagine that during wakefulness insular cortex receives those visceral signals which are important for organization of active behavior. For example, this can be feeding behavior, which includes chewing, swallowing and analysis of the food content by chemo- and mechanoreceptors located in the mouth. The brain uses this information in order to tune gastrointestinal tract for optimal digestion of particular food content as well as to adjust feeding behavior. Other types of consciously controlled “visceral” behaviors are defecation and urination, which play an important role also in animal communication in natural conditions. In addition, in wakefulness insular cortex can monitor and drive cardio-respiratory activity that requires constant adjustment to the current needs of a behaving animal. May be because of that activities related with these functions dominate in the insular cortex (Uddin et al., 2017). However, during sleep afferentation to the insular cortex may still originate from the visceral systems, but now most likely it would be related to the state of those organs. This information will be used for diagnostic, and, in the cases of detected deflections from normal conditions, for recovery of their functionality. Obviously, destination of the efferent insular projections in sleep should be different as well in order to support such difference in function.

If effective afferentation and efferentation of the insular cortex change between sleep and wakefulness, one would expect that properties of the insular neuronal responses to exteroceptive and interoceptive stimuli would also change with a transition from wakefulness to sleep, as it had happened in our investigations of other cortical areas.

In this study, focused on investigation of the relationship of the insular cortex with gastro-intestinal system, we aimed to test which of these two alternative hypotheses describes organization of the insular cortex better. We recorded local field potentials and neuronal spiking activity from the insular cortex and investigated their responses to electrical stimulation of intestine applied in wakefulness and in sleep. In addition, we compared effects of electrical stimulation of the insular neurons on orofacial muscle movements and intestinal motility in these two states of vigilance.

## Materials and Methods

### General Approach

Results presented in this study were obtained in experiments performed with two adult female cats. All experiments with these cats were conducted using our modification of the painless head fixation approach (Noda et al., 1971; Pigarev et al., 2009), designed to provide stable recordings of neuronal activity and other visceral parameters during the course of long (up to 6 h daily) chronic experiments in big animal models. Recording sessions covered periods of both wakefulness and sleep. Experiments were conducted during daytime.

Surgery and treatment of the animals were carried out in accordance with the Ethical Principles for the maintenance and use of animals in neuroscience research (Zimmermann, 1987), NIH guidelines for the care and use of animals and Declaration of Helsinki on Ethical Principles for Medical Research.

Current Russian laws do not bind scientific Institutes to have special Ethic committees; instead, assessment of the ethical components of a research proposal is conducted by the Institutional Scientific Councils. According to the practice of the Russian Foundations distributing funding for scientific grants, the Council of Reviewers prior to making a decision regarding financial support of a particular study performs ethics evaluation. Both Councils are guided by the recommendations of the above-mentioned documents.

Preparation of animals to the experiments included a habituation of each animal to the laboratory environment and to the investigators working with these animals. This “shaping” process, which was based on positive reinforcement techniques, usually took a couple of weeks before performing any surgery. Cats were fed each day in the laboratory space to minimize anxiety associated with the experimental conditions. Each animal was fed 50 g of standard wet cat food 30 min prior to every recording session in order to observe postprandial pattern of intestinal activity. In addition, this protocol reduced potential fasting-associated anxiety and promoted sleepiness to be able to record enough data in sleep state. The rest of the food was given after the recording session.

### The Surgeries

Two separate surgical procedures were performed with each cat under general anesthesia (premedication with Xylazine 0.15 ml/kg, and anesthesia with Zoletil, first injections 6 mg/kg, and additional doses of 5 mg periodically with intervals about 20 min to keep the appropriate level of sedation).

During the first operation, performed with the animal's head fixed in a stereotaxic apparatus, we attached the “halo” type frame to the skull of the animal. After the frame was fixed, relevant stereotaxic coordinates were transferred to the surface of the frame in order to guide further microelectrode penetrations. During this surgery two electrodes for recording of the general EEG signal were implanted as well. These epidural elgiloy electrodes were placed under the skull bone through small drilled holes (2 mm) located above frontal and occipital cortical areas, and stabilized by acrylic dental cement.

In brief, the first surgery included removal of soft tissues from the dorsal head surface and insertion of eight small screws into the skull at ~15 mm distance right and left from the midline. The screws were connected with thin wire that served as a frame, the frame was reinforced with dental acrylic cement, and the exposed top surface of a skull was covered with a thin layer of the same cement. This frame allowed head fixation in a special head holder for recording stability and partial restriction of animal movements during experiments. However, animal could change position of the body and after short habituation stayed for a long time in experimental setting without any signs of discomfort. The head fixation training was commenced only after 2 weeks recovery period following the first surgery. Duration of this training depended on individual behavior of an animal. Usually within a couple of weeks an animal becomes acclimatized to the head fixation and begins sleeping with its head fixed. After that the second surgery can be performed.

During the second operation we implanted intramural bipolar silver electrodes (interelectrode distance ~2 mm) into the wall of duodenum in order to conduct both the recording of intestinal myoelectrical activity and the electrical stimulation of the intestinal wall. Intramural electrodes were implanted using the method proposed by Papasova and Milenov (1965) and Papasova et al. (1966). Details of this procedure were also previously described in Pigarev et al. (2013). This type of electrodes due to very short interelectrode distance allowed recording of intestinal myoelectric activity while minimizing possible influence of motion artifacts related to normal postural changes in wakefulness. However, these electrodes have limited lifespan (up to 3 months) because of the wear and tear resulting from intestinal motility and animal's movements, and this time defined the total length of the experiments with each animal. We usually implanted two pairs of such bipolar electrodes distanced 2 cm from each other, and selected for recording the pair demonstrated better signal quality.

### Recording of Neuronal Activity and Electrical Stimulation of the Insular Cortex

In this study we simultaneously recorded neuronal activity from right and left insular cortices. Electrodes were introduced through two drilled holes (2.5 mm in diameter) via conical plastic guiding tubes. This method was initially developed for conducting chronic experiments in monkeys (see Pigarev et al., 2009 for the details); however, later we began to apply this approach to experiments in cats (Pigarev et al., 2013).

The plastic guiding tubes were positioned above the expected location of the portion of the insula that possesses visceral projections (see Clascá et al., 2000). Localization and verification of these positions is described in details in the section titled “The use of MRI imaging for precise stereotaxic microelectrode positioning within particular anatomical locations in a brain.”

Neuronal activity was recorded using custom-manufactured tungsten varnish-coated microelectrodes with 0.5–1 mΩ impedance. Electrodes were first varnished up to the complete lack of 1 kHz current propagation. After that, their tips were opened using high voltage electrical micro discharges through the air gap, which melted the tungsten at their sharp tips and transformed them into small balls of 5–10 μm in diameter. This procedure preserved sufficiently thick layer of varnish insulation just near these balls excluding capacity leakage of high frequency signals.

In this study we used “bipolar” recording between two microelectrodes. The tips of these parallel electrodes were positioned at 300–500 μm distance from each other. This type of recording lets us (1) maximize the cell count per recording session; (2) record local field potentials with local reference; (3) use the same microelectrodes to deliver local microstimulation to approximately the same areas of the insular cortex where neuronal activity was recorded from (see details below). Both microelectrodes were moved along the vertical axis together by a custom-made lightweight mechanical microdrive fixed to the head implant. This microdrive was attached to the head frame for the entire period of recordings with a single pair of microelectrodes. Such period could last for up to several weeks. Before every new recording session, we moved electrodes at least 200 microns down to the new group of neurons.

Neuronal activity was amplified in 0.3–2,000 Hz frequency range by NeuroBioLab amplifier designed for bipolar recording with two high impedance microelectrodes. This amplifier had two input head stages. Signals were sampled at 10,000 Hz, stored and offline filtered in 0.3–250 Hz range for further analysis of local field potentials and in 300–4,000 Hz range for analysis of spiking activity.

Stimulation of the insular cortex was applied via the same two electrodes used for recording of cortical activity. During stimulation microelectrodes were disconnected from the input of the amplifier, and connected to the output of NeuroBioLab constant current stimulator. This reconnection was done by custom-made electromechanical device. During this procedure amplifier was temporary saturated, and returned to the working range only after being connected again to the recording electrodes. Because of the wide frequency range used in our study, this return took some time, during which we did not have signals from the insular neuronal activity. However, after the off-line processing included subtraction of an averaged artifact this interval of signal distortion could be reduced up to 12 ms. Recording of neuronal activity from each new cortical site was always conducted first, followed by stimulation. It seems important to note that in this study only a very small area of the cortex was stimulated as the current traveled between the tips of the electrodes located at 300–500 μm distance from each other.

Monophasic stimulation was applied in 40 ms trains of 1 ms pulses at 200 Hz and current within a range 15–100 μA, adjusted for every recording site to produce small amplitude brief orofacial movements in wakefulness but no movement in sleep. Duration of interstimulus intervals for stimulation in each hemisphere was 60 s for Cat 1 and 120 s for Cat 2.

The main criteria for sleep quality during sessions involving microstimulation of the insular cortex during sleep was the absence of motions (usually orofacial and of small amplitudes) evoked by the same stimulation in wakefulness. However, in addition to that, off-line comparison of EEG power spectral density before and after stimulation was performed to confirm undisturbed sleep (see below).

### Recording of the Myoelectrical Activity and Electrical Stimulation of the Duodenum

For recording of the duodenal myoelectrical activity implanted electrodes were connected to the input of NeuroBioLab amplifier (0.1–40 Hz frequency range), and signals were stored on a hard drive with 200 Hz sampling rate.

The same electrodes were used for electrical stimulation of the duodenum. For stimulation these electrodes were reconnected to the output of NeuroBioLab stimulator that provided constant current stimulation. Local monophasic stimulation was delivered in 20 ms trains of 0.5 ms square pulses at 200 Hz and 150 μA, with 45–60 s interstimulus intervals. The reconnection of the electrodes to stimulator was performed by custom-made electromechanical device similar to that used in recording and stimulation of the insular cortex.

During stimulation recording was stopped, and because of temporal saturation of the amplifier, and its subsequent recovery to baseline level myoelectrical activity of the duodenum was not recorded during period of stimulation (20 ms) and immediately after (20 ms). However, this was not critical for our study because this interval was definitely shorter than the time of propagation of the nervous signal from intestine to the insular cortex.

Parameters of electrical stimulation of the duodenum were titrated to the levels that did not disturb sleep of an animal. The quality of sleep after intestinal stimulation was initially tested on-line by observation of animal's behavior (video monitoring) and later off-line by comparing EEG power spectral density before and after stimulation (described in details below). While electrical stimulation of duodenum was done in both Cats, the insular stimulation was performed mostly to analyze behavioral responses in Cat1, and to observe both behavioral and myoelectrical activity changes in Cat2.

### General Vigilance State Monitoring

We aimed to compare various neuronal and myoelectric effects occurring in either sleep or wakefulness. In order to separate these states of vigilance standard polysomnographic parameters were recorded.

Electrocardiogram (ECG) was recorded between one electrode in the wall of duodenum and the other electrode—epidurally inserted stainless screw in the middle of the dorsal surface of the skull, which was always used for grounding of the animal during electrophysiological recordings. ECG signal was sampled at 1 kHz.

The respiration rate was estimated from respiratory movements (recorded using thoracoabdominal belt with a piezoelectric sensor) and nasal air flow (with thermo-sensors placed in front of a nose). Sampling rate was 200 Hz.

Eye movements and opening/closing of the eyelids were recorded with an infrared oculometer with 200 Hz sampling rate.

Permanent video recording of the animal's head and shoulder area was synchronized with recordings of all the other physiological parameters.

Duration of a single recording session ranged from 2 to 5 h. The number of stimulations per sleep or wakefulness state depended on the duration of each state and was variable without a systematic difference between states (*p* = 0.1308, Wilcoxon Signed Rank Test). Responses to stimulations were not included into analysis if they coincided with motion artifacts; in addition, we excluded from further analysis all stimulation trials where sleep/wakefulness state changed during the trial. Resulting amount of stimulation trials (pooled for both cats) included into analysis of neuronal activity ranged from 17 to 73 in sleep and from 18 to 59 in wakefulness per recording session.

### The Use of MRI Scans for Precise Stereotaxic Microelectrode Positioning Within Particular Anatomical Locations in a Brain

In long-lasting chronic experiments individual recording localization cannot be marked with electrolytic lesions common for acute and short chronic experiments. These lesions do not remain detectable in the brain tissue for a long time. In addition, ethical considerations prevent us from sacrificing animals after experiments if it can be avoided. However, there is an alternative method for localization of the recording places based on stereotaxic approach. The cats are known as good animals for stereotaxic localization due to relatively constant size of their brains, and a very reliable stereotaxic atlas for cat's brain is available (Reinoso-Suárez, 1961). However, even in cats variations of the general size and the sulci patterns were described (see e.g., Otsuka and Hassler, 1962).

That is why, for electrode penetration precision one would need to have stereotaxic maps created individually for each animal. For this purpose, before the experiments both animals were MRI-scanned in 1.5 Tesla scanner. Using that MRI information such individual maps were constructed for each of two cats. These individual maps allowed performing more precise stereotaxic penetrations into the desired locations of the insular cortex. Analysis of the MRI scans for the purpose of these experiments included several steps.

**Figure 1:**
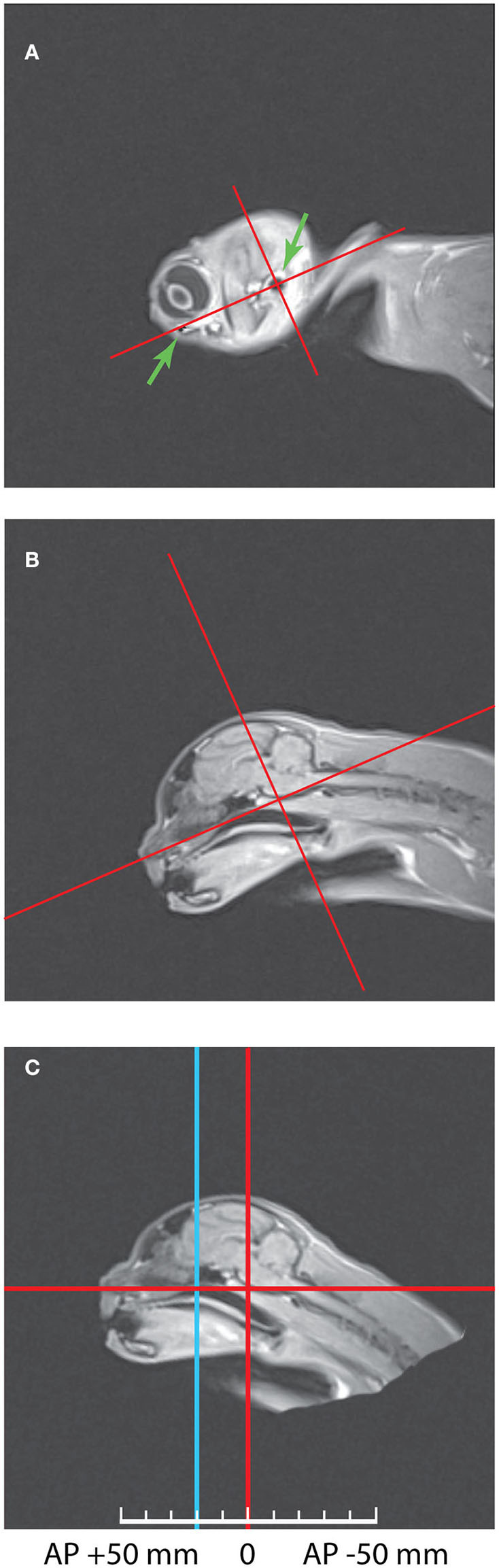
The method of combining Horsley—Clarke stereotaxic planes with MRI scans of a cat's head. **(A)** Sagittal MRI scan of the lateral border of the Cat 1 skull. The arrows mark two points to construct the horizontal axis: acoustic meatus and infraorbital margin. Red lines show horizontal and vertical axes of the Horsley-Clarke stereotaxic planes. **(B)** Medial sagittal scan before MRI data recalculation. **(C)** Medial sagittal scan after MRI data recalculation to the normal orientation. Blue line represents coordinates AP + 20. Further details of the procedure are provided in the text.

First, it was important to combine these scans with the stereotaxic coordinate system of Horsley—Clarke. The best way to do so was to scan the heads placed into plastic stereotaxic frame with water marks of zero stereotaxic planes, as it was done e.g., in our previous study in monkeys (Pigarev et al., 1997). However, in cats the main markers determining location of the zero planes in Horsley-Clarke stereotaxic system for cats (acoustic meatuses and infraorbital margins) are perfectly visible on MRI scans, allowing establishing stereotaxic zero planes with the necessary precision. Figure 1 illustrates this procedure. Panel (A) of this figure shows the sagittal MRI section with the most lateral part of the brain of Cat 1, with green arrows indicating the infraorbital margin (left arrow) and acoustic meatus (right arrow), both structures can be seen as dark elements of the picture. The line connecting these two points creates the horizontal Horsley-Clarke plane. Orthogonal line crossing acoustic meatus creates the vertical plane. In panel (B) these stereotaxic axes were projected on the middle sagittal section of the brain. It is seen that during the scanning procedure the head of that cat was tilted downwards. Panel (C) demonstrates the same scan after the imaging data were recalculated in order to bring the head to the normal stereotaxic orientation. This procedure allows obtaining the usual stereotaxic planes of the brain for any antero-posterior coordinates. Vertical blue line shows location of AP +20 plane, which is also shown in the next figure featuring the coronal section. In Cat 2 head orientation during scanning was practically in proper stereotaxic orientation.

After the vertical zero planes were established, we measured the individual brain sizes for each cat. In our case Cat 1 had standard antero-posterior dimension of the cat brain-−40 mm. However, location of the brain itself was shifted 2 mm backwards relative to vertical stereotaxic zero plane of the atlas. The brain of Cat 2 was 1 mm smaller than of a “standard” cat and was shifted 1 mm backwards (this is not shown in the figures).

At the next step we searched for the locations of the main sulci in the region of interest. Localizations of the cortical areas, although variable for different animals, are known to be more strongly related to anatomical features such as sulci and gyri, than to stereotaxic coordinates themselves. Localization of sulci on the brain surface can be facilitated with the use of “dynamic” stereotaxic atlas of the brain allowing to move between the sections to trace anatomical structures. We created such dynamic atlas using the regular atlas of a cat brain (Reinoso-Suárez, 1961). Since MRI visualization softwares always have the option of dynamic structure examination, combining these two resources makes it much easier to localize the corresponding positions of the important anatomical structures (e.g., sulci) for each animal. MRI locations of sulci on the brain surface are often highlighted by bright spots because blood vessels are localized along the sulci, and local increase of water concentration is seen on MRI scans as bright spots.

**Figure 2:**
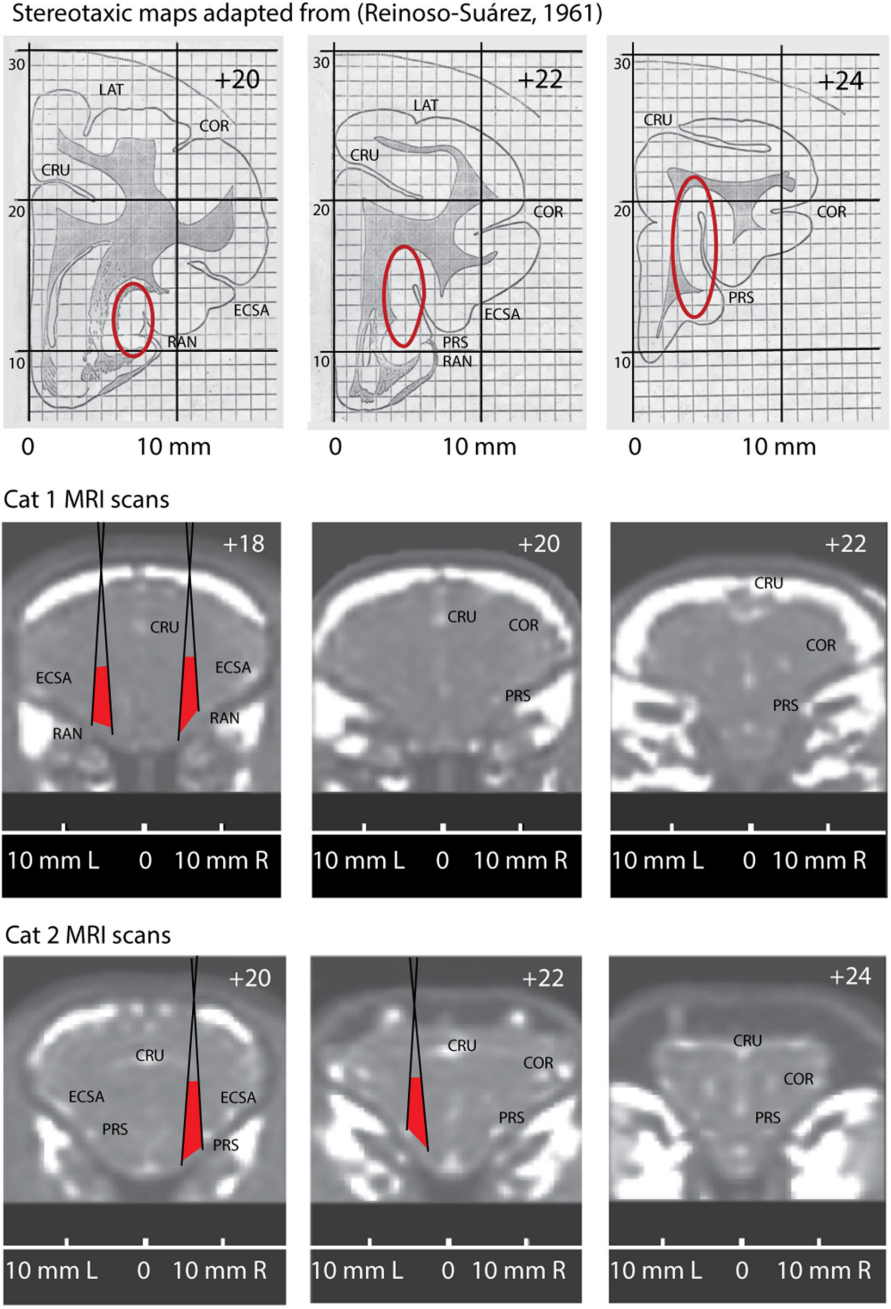
Comparison of the stereotaxic maps adopted from stereotaxic atlas of a cat brain (Reinoso-Suárez, 1961) with anatomically corresponding MRI scans of Cat 1 and Cat 2. Top row. Three stereotaxic maps for positions AP +20, AP +22 and AP +24. Red ovals indicate expected location of the insular cortex. Two bottom rows demonstrate three anatomically corresponding coronal MRI slices through the brains of Cat1 and Cat 2, with their individual stereotaxic coordinates. Positions of the conic recording tubes located in right and left hemispheres of these cats are superimposed on the scans. Red areas indicate the regions potentially covered by the microelectrodes. CRU, Sulcus Cruciatus; ECSA, Sulcus Ectosylvius Anterior; PRS, Sulcus Praesylvius; RAN, Sulcus Rhinicus Anterior; COR, Sulcus Coronarius.

We present key elements of this procedure in Figure 2. The top row shows three fragments adopted from the stereotaxic atlas (Reinoso-Suárez, 1961) with red ovals indicating expected locations of the insular cortex (Clascá et al., 2000).

Below that we present the corresponding coronal section of the MRI scans of Cats 1 and 2. Note that the obtained stereotaxic coordinates of the insular cortex differ between the cats because of the above mentioned differences of the brain proportions and localization relative to the cranial bones (due to the variations of bone structures and head sizes between animals). On the presented MRI scans one can see the above described small bright spots marking anatomical localizations of the sulci, which are labeled on the brain surface.

As it mentioned above, we used conical guiding tubes for microelectrodes penetrations. These tubes are schematically superimposed on the scans and the maximal angles of microelectrode scatter are shown by oblique black lines. Red areas between these lines demarcate the regions our recording sites were confined to.

Using these individual stereotaxic coordinates recording electrodes were directed to the anatomically defined parts of the brain where insular cortex is expected to be localized.

However, in addition to the described procedure, initial positioning of the electrodes' tips within the insular cortex was functionally verified using independent criteria. The verification relied on known effects of electrical microstimulations of this cortical area in previous studies of the insular cortex (Olsson and Landgren, 1980; Iwata et al., 1985; Caruana et al., 2011). These effects of microstimulation will be presented in the corresponding section of the results.

## Data Analysis

### Analysis of the Insular Responses to Electrical Stimulation of Duodenum in Sleep and Wakefulness

Data sets from every animal were analyzed separately, and for each animal every recorded neuronal activity from every recording site were also analyzed individually. Presented below effects of duodenal stimulations on local field potentials and activities of individual neurons were consisted between two animals.

### Local Field Potentials

As mentioned above we recorded neuronal activity from the insular cortex using two microelectrodes with a distance between their tips ranging from 300 to 500 μm. Using the amplifier with the wide frequency range (0.3–2,000 Hz) we recorded changes of slow potentials–the local field potentials, as well as spiking activity. This method allowed us to be sure that recorded activity was generated in the close vicinity of our electrodes (Herreras, 2016). Recorded signal was first filtered in 0.3–250 Hz range for the analysis of local field potentials. To the best of our knowledge, intracortical event-related potentials to intestinal stimulation in non-anesthetized animals have not been described before, although individual insular cells responding to visceral stimulation were observed (Cechetto and Saper, 1987). We applied the logic, common to the studies of event-related potentials (Kappenman and Luck, 2011), to our analysis of the local field potentials.

The emergence of an event-related response requires summation of postsynaptic potentials simultaneously occurring in many neurons. The presence of the evoked response would therefore indicate the arrival of an afferent signal to the cortical area. Thus, as a first step we estimated the presence of an evoked response in sleep and wakefulness using Residual Orthogonality Test (Achim et al., 1988; Picton et al., 1995). It allows testing if a post-stimulus deflection of amplitude contains a specific response or just occurs randomly. That involves de-trending of a recorded signal and obtaining sums of all possible pairwise point-to-point multiplied signals coming from all trials within a particular interval. Either positive or negative response consistent between individual trials would result in a positive sum while values for randomly occurring deflections would be randomly distributed around zero. Thus, significant positive mean of all possible sums indicates the presence of a response. The test was performed for 500 ms interval starting 40 ms after stimulus onset, and the mean of the resulting values was compared to zero with the *T*-test.

In addition to that, for the cases where significant evoked potential was observed, we compared its maximal amplitude between sleep and wakefulness after z-scoring signals to the pre-stimulus level (500 ms interval before stimulus onset) individually for each trial, as amplitudes of local field potentials in slow wave sleep might be higher as a result of delta-wave activity, and that can affect comparisons. Mean amplitudes at 40 ms interval surrounding corresponding response maxima were compared between sleep and wakefulness using Wilcoxon Rank Sum test.

### Neuronal Spiking Activity

Using bipolar recording made from two closely spaced microelectrodes we could simultaneously register activity generated by neurons located near one and the other electrode. With wide frequency range amplifiers (0.3–2,000 Hz) we frequently saw that recorded spikes were indeed of different polarities. In addition, from each electrode we usually recorded spikes generated by several neurons, as it is common for extracellular recordings. The spiking activity was band-pass filtered (300–4,000 Hz) and analyzed by the Spike 2 (Cambridge Electronic Design Limited) inbuilt algorithm of spike sorting based on amplitude triggering and waveform matching, and spikes from different neurons were stored in different channels as events on time scale.

**Figure 3:**
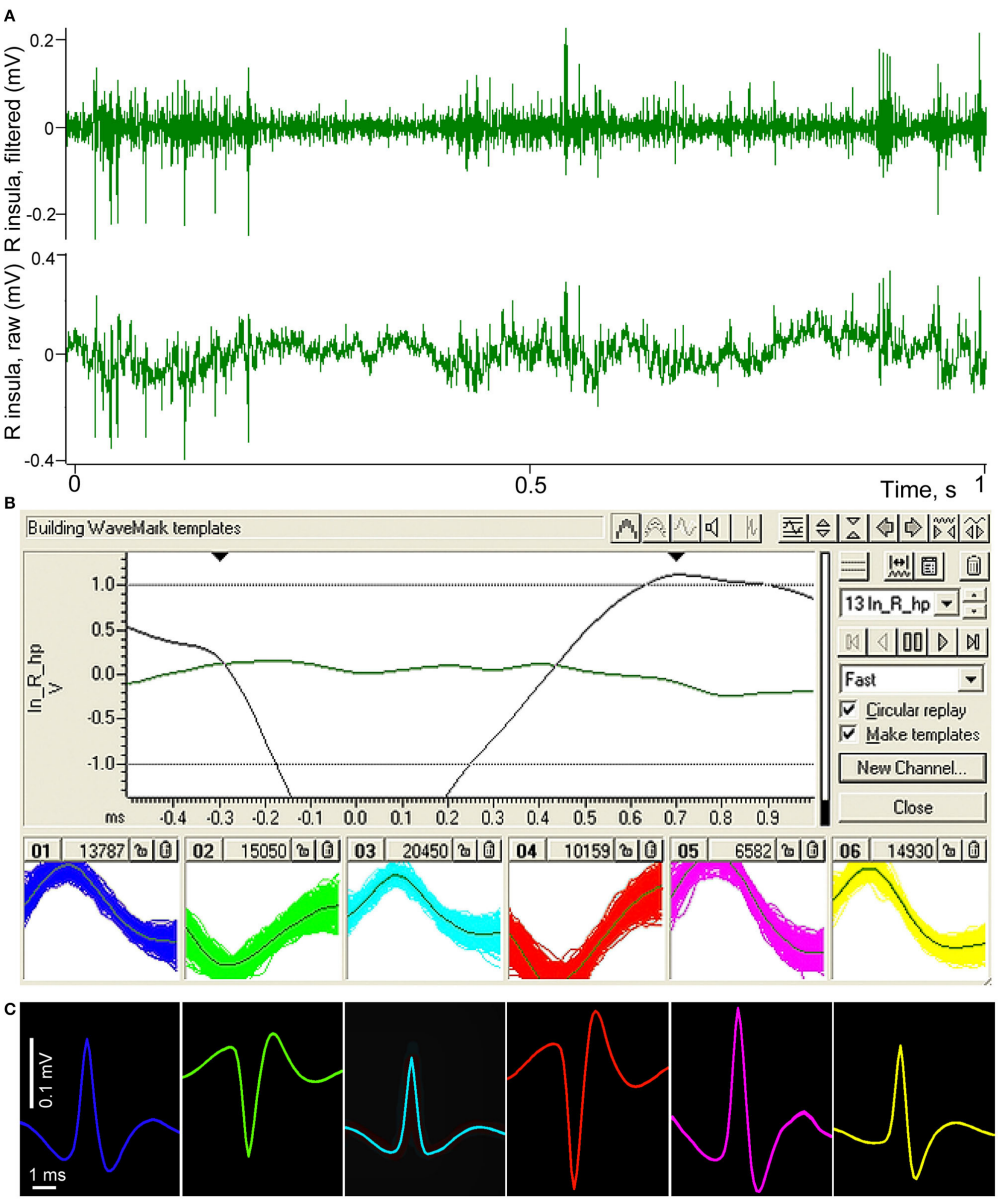
Illustration of spike sorting procedure performed in Spike 2 (CED) software. **(A)** Fragment of a raw recording of neuronal activity of the right insular cortex conducted using two microelectrodes with about 500 microns distance between their tips (lower green channel). The same fragment after band-pass filtration, demonstrating high frequency spiking activity (upper green channel). **(B)** Spike 2 templating window with investigator-defined parameters of templating on top, bottom part of the panel shows six selected groups of spikes sorted by their amplitudes, polarity and shape; the individual spikes are superposed on their templates. **(C)** Resulting averaged shapes of the same selected spikes, presented in the same color.

Example of the spike sorting procedure is demonstrated in Figure 3. In top panel (A) of the figure a short fragment of raw recording of the right insular area is shown as the bottom channel, and the result of band-pass filtration where only high frequency activity such as neuronal spikes remains in the signal is shown as the top channel. Panel (B) demonstrates the window from Spike 2 program with investigator-defined parameters of templating on top of the panel. Below, in six small windows, resulting sorted spikes are superposed on the templates. In panel (C) these resulting spikes of different neurons are averaged for the entire length of recording, separately for every neuron using the filtered signal, triggered by each of the corresponded templates. One can see that the so “templated” neurons can be easily distinguished from each other by their shape, amplitude and polarity. Polarity differences indicate that four of the spikes were most probably generated by neurons located near one electrode of the pair, and two other neurons—near the second electrode. At the same time, all these neurons were located within a small cortical volume not exceeding 700 microns.

At the next step spiking events were smoothed with Gaussian kernel of υ = 20 ms (Wallisch et al., 2010), and thus were transformed into the curve represented spike frequency rate changes in time.

Neuronal responses to intestinal stimulation were averaged separately for sleep and wakefulness conditions. Following the criteria suggested by Reed et al. (2011), responses were considered significant if they deflected above or below three standard deviations (SD) of the baseline activity calculated in 500 ms before the onset of microstimulation, and remained consistently deviated from the baseline by at least 2 SD for at least 30 ms within 40–500 ms interval after the onset of stimulation. Interval of stimulation and immediately following the stimulation was excluded from the analysis in order to avoid possible influence of electrical artifact of stimulation.

### Analysis of Duodenal Responses to Electrical Microstimulation of the Insular Cortex in Sleep and Wakefulness

Considering that insular cortex is known to possess motor areas involved in regulation of various visceral functions including digestive tract motility (Penfield and Faulk, 1955; Neafsey, 1990; Stephani et al., 2011; Nieuwenhuys, 2012), we analyzed responses of intestinal myoelectric activity to insular stimulation. Myoelectric activity of small intestine represents only a small fraction of potentially changing parameters of the visceral activity that can result from such stimulation. They can be not only motoric, but involve various metabolic functions, and these changes most probably are not limited to small intestine. However, the current state of the field does not allow for most of these functions to be repeatedly recorded in non-anesthetized animal without causing significant adverse effects and discomfort to the animal. With the additional restriction of preserving sleep quality and local application of stimulation, effects of such stimulation are not expected to be omnipresent or particularly large in amplitude. Our goal was to compare their detectable presence between wakefulness and sleep states to assess impact of the insular cortex in these states.

Myoelectric activity of small intestine can improve the chances to register stimulation-induced visceral activity as not only the small intestine itself can respond to such stimulation, but in addition to that stomach responses are expected to cause changes in intestinal activity. Thus, if a particular area of the insular cortex is involved in regulation of either intestinal or stomach contractile activity, one might be able to detect evoked responses despite recording only from the intestinal wall.

As visceral responses often need prolonged intervals to develop and therefore have more chances to be affected by motion artifacts, recordings were visually inspected, and trials where the signal in question was affected removed.

Myoelectrical activity of the upper part of small intestine (duodenum) is normally periodic and contains simple waves (sometimes called Electrical Control Activity) with very constant slow rhythmicity, and also high frequency spike bursts, or spike-potentials (Electrical Response Activity), which are irregularly superimposed on some of the simple waves. Simple waves reflect activity of the enteric nervous system and therefore determine the possibility and the timing of contractions, while faster spike-potentials are more tightly related to the actual occurrence of contractions of the intestinal wall (Papasova and Milenov, 1965; Papasova et al., 1966; Costa and Furness, 1982; Sarna, 1989; Martinez-de-Juan et al., 2000). Although simple waves are originated from a pacemaker activity within the enteric nervous system, their amplitude is modulated by parasympathetic and sympathetic influences, as well as distension level of the particular part of intestine and some gastrointestinal hormones. The effectiveness of these arriving influences depends on the phase of the simple wave activity (for a detailed review see Costa and Furness, 1982). Postprandial period of increased and irregular motor activity of intestine usually lasts for several hours after an intake of a caloric meal, thus covering the whole period of our recording session. During this time contractions occur irregularly at an approximate rate of 4–5 per min.

In both humans and small animals, the energy of duodenal simple waves spectrum is concentrated below 2 Hz while for the waves with spike-potentials it is ranging depending upon the way of recording but usually is substantially concentrated between 2 and 10 Hz (Sarna, 1989; Martinez-de-Juan et al., 2000; Garcia-Casado et al., 2005, 2006). Thus, we compared power spectrum density of intestinal activity in 40 s intervals before and after stimulation in simple wave frequency range (0–2 Hz) in sleep and wakefulness using Wilcoxon signed rank test. The post-stimulus 40 s period of the analysis started 0.5 s after the stimulus onset. We also analyzed spike-potential signals (3–10 Hz) within 20 s interval centered at the maximum of the simple wave modulation after the stimulus onset in comparison to the 20 s interval located at the same distance before the stimulus onset.

Multitaper spectral analysis was conducted using 5 Slepian tapers and a time bandwidth product equal to three using Chronux toolbox for Matlab (Mitra and Pesaran, 1999; Jarvis and Mitra, 2001; available from http://chronux.org/).

For the recordings were significant response was detected we conducted sleep disturbance test using EEG signal similar to described below for the intestinal stimulation. No sleep disturbances were detected.

## Results

### Slow Wave Sleep Characterization

Comparison of neuronal activity between sleep and wakefulness conditions requires clear separation of these two states. The initial state separation was based on visual inspection of the polysomnograms and video recordings. The usual signs of sleep were used at that stage: an increase of EEG slow wave amplitude, slowing of respiration, decrease of heart rate variability, general decrease of animal's motor activity, slow gaze drifts replacing saccadic movements, closing of the eyelids. After selecting intervals of sleep and wakefulness using these criteria, we performed quantitative comparison of power spectral density between two conditions. We analyzed EEG spectral compositions within frequency ranges known to be sensitive to the state of vigilance: delta (1–4 Hz), spindles (7–14), and gamma (30–75 Hz) frequency ranges. Sleep is characterized by increased delta and spindle activity while increased gamma is common for wakefulness (Contreras and Steriade, 1996; Destexhe et al., 1999). EEG spectra were obtained using multitaper spectral analysis using 5 Slepian tapers and a time bandwidth product equal to three (Mitra and Pesaran, 1999; Jarvis and Mitra, 2001). We found that in all recordings in both cats delta and spindle range power spectral density values in EEG were significantly higher in sleep while power in gamma range was always higher in wakefulness (Wilcoxon Rank Sum test *p* < 0.001).

### Test for Potential Sleep Disturbance Caused by Electrical Stimulation of Small Intestine

**Figure 4:**
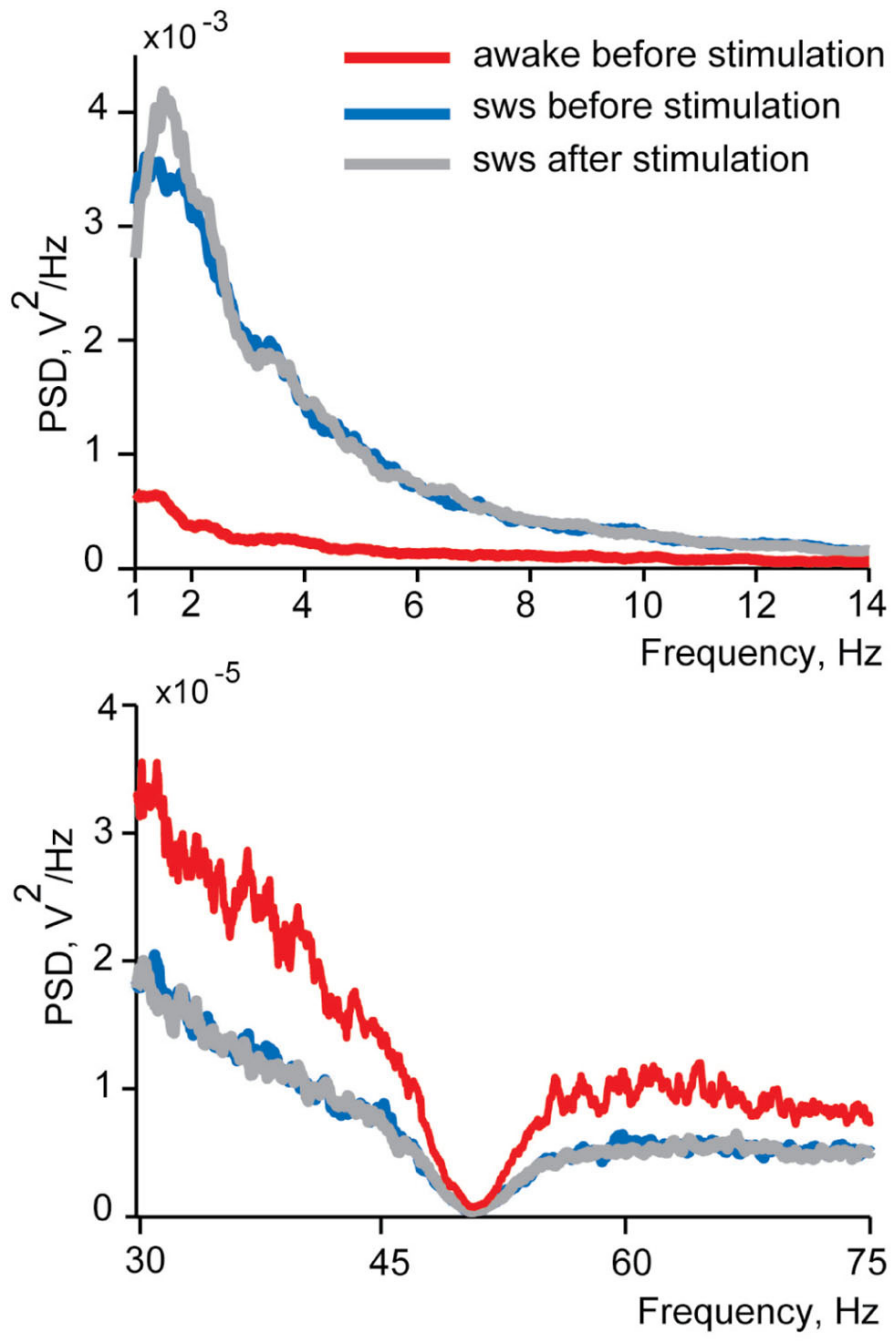
An example of power spectral density functions of EEG during wakefulness and in sleep before and after intestinal electrical stimulation for one recording session. Upper panel demonstrates delta and spindles frequency range and lower panel—gamma frequency range. Red lines show power spectral density in wakefulness from 10 s intervals before each stimulation, blue lines represent spectrums of sleep before stimulation and gray lines—spectrum in sleep after the stimulation. Note the different vertical time scales in upper and lower panels. The dimpling of all curves in the lower panel around 50 Hz is caused by the use of a notch filter.

In this study we investigated LFP and spiking activity changes in the insular cortex in response to electrical stimulation of small intestine delivered in sleep and wakefulness. Therefore, it was crucial to ensure that stimulation delivered during sleep did not wake a sleeping animal up. In case of an awakening of a cat by stimulations we would expect to see decreased delta and spindles power and/or increased gamma power of the EEG spectra. To test for the sleep disturbance, we compared power spectral density in these ranges between the 10 s EEG intervals before and after every intestinal stimulation for each recording session. The comparisons were made within the frequency ranges identified as sensitive to the state of vigilance during slow wave sleep characterization (delta, spindles, and gamma). Changes of the sleep quality are expected to manifest as differences in spectral composition after the stimulation corresponding to awakening—power decrease in delta and spindles frequency ranges and increase in the gamma range. We did not find any such awakening effects in any recordings of both cats in sleep (Wilcoxon signed-rank test *p* > 0.05), and concluded that our stimulation did not cause sleep disturbance detectable with EEG. No behavioral signals of awakening (body or eye movements) were observed as well. Figure 4 demonstrates spectra before stimulation, for wakefulness and sleep, as well as after stimulation in sleep.

Similar analysis performed for the awake condition revealed that for two recording sessions (out of 30) stimulation during wakefulness led to a decrease in gamma power after the stimulation, in one recording sessions to an increase in delta power and in one to an increase in spindles (Wilcoxon signed-rank test *p* < 0.05). Thus, if there is any effect of stimulation on the level of vigilance it might be some mild somnogenic influence.

In addition, analogous categorization of vigilance state and sleep disturbances by electrical intestinal stimulation were conducted using recorded LFPs since local sleep dynamics might differ across the cortex (Pigarev, 1997). Patterns of differences in LFP activity between sleep and wakefulness were similar to the ones observed in EEG in delta and spindles frequency ranges (Wilcoxon Rank Sum test, *p* < 0.05). However, gamma activity demonstrated no such consistency, with all possible variants of differences between states of vigilance (Wilcoxon Rank Sum test), no difference between conditions (*p* > 0.05), or positive or negative differences (*p* < 0.05) in different recording sites. This result is in accordance with a body of data describing the presence of intracortical gamma in sleep (Le Van Quyen et al., 2010) as well as with our assumption that processing of the incoming visceral information by the cortical networks continues in sleep.

Within the ranges of delta and spindles no awakening effects of stimulation were observed in LFP as well (Wilcoxon signed-rank test *p* > 0.05). On the contrary, we found that in four cases in wakefulness and four cases in sleep stimulation led to significant increase in delta power (*p* < 0.05). Thus, as for the global EEG, with the intracortical LFP we did not find any awakening effects but rather observed some indications of mild somnogenic effects of applied stimulation, as it was previously reported for intestinal stimulations (Kukorelli and Juhasz, 1977; Pigarev, 1994).

The absence of sleep disturbance after stimulation allowed us to move to the next step of data analysis—the investigation of the insular LFP responses to electrical stimulation of small intestine.

### Event-Related Local Field Potentials in Response to Electrical Stimulation of Small Intestine

Evoked LFP activity in response to stimulations applied in both wakefulness and sleep was collected from 14 recording sites in Cat 1 and 16 in Cat 2, respectively (total *N* = 30). Responses to intestinal stimulation were found in 15 of 30 recording sites in sleep (nine in Cat 1 and six in Cat 2) and six of 30 in wakefulness (four in Cat 1 and two in Cat 2) (Residual Orthogonality Test, Achim et al., 1988). Thus, proportion of responsive sites in sleep was significantly higher (*p* = 0.015, Fisher exact test). Note that recordings in both states were made from the same cortical sites and with the same stimulation parameters.

**Figure 5:**
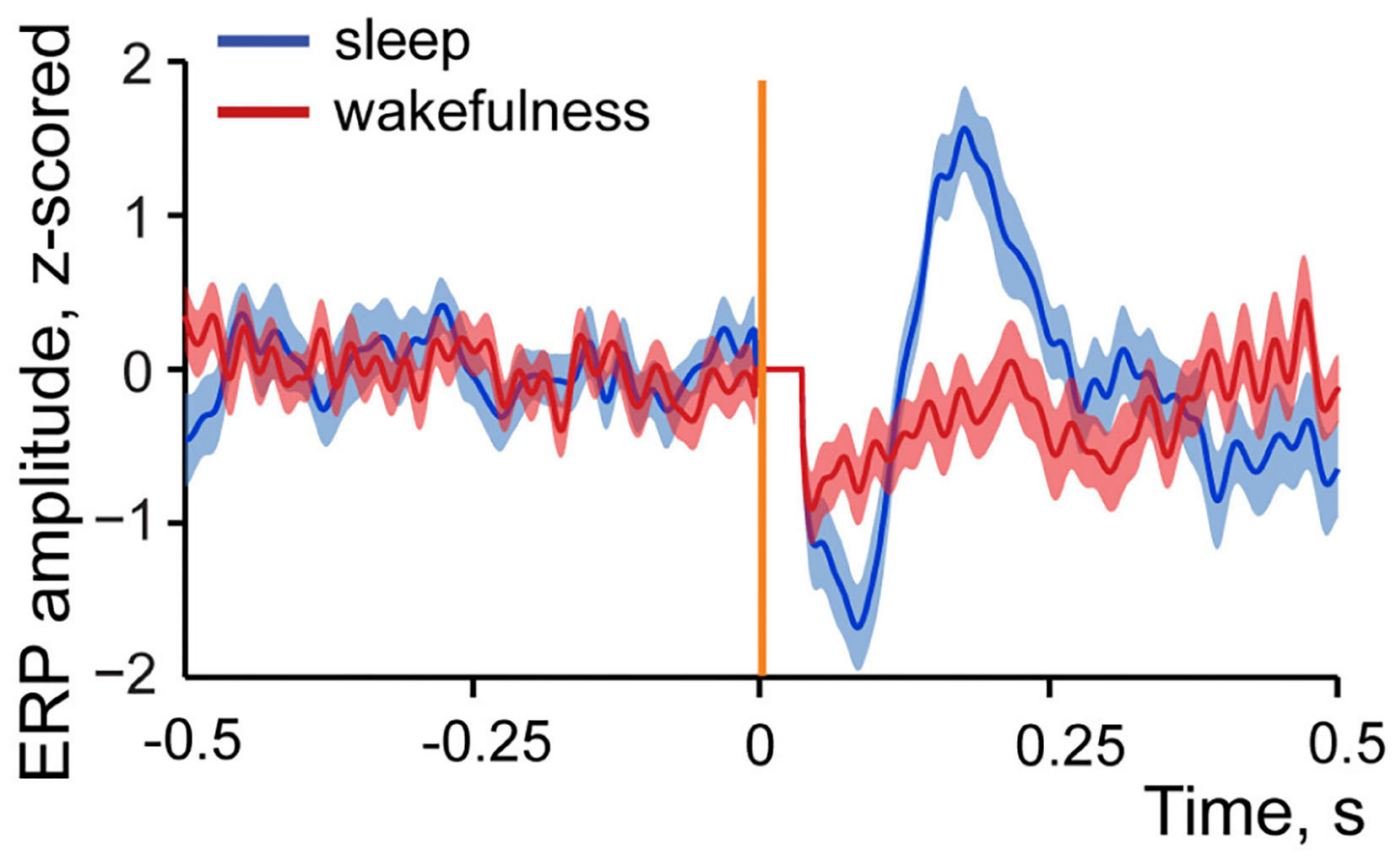
Example of an averaged event-related local field potential recorded in the insular cortex in response to intestinal stimulation in sleep and wakefulness. Dark blue line—z-scored response to intestinal stimulation in sleep. *N* stimulations = 29. Dark red line—z-scored response to intestinal stimulation in wakefulness. *N* stimulations = 39. Standard errors of mean are shown in light blue and light red colors. Vertical orange line indicates time of stimulation onset, artifact of stimulation is removed (horizontal line after stimulation onset).

Figure 5 demonstrates z-scored averaged event-related potentials to intestinal stimulation in sleep and wakefulness. One can see that amplitude of the response is considerably higher in sleep in contrast to wakefulness. This state-dependent difference was typical for all studied evoked responses, and the maximum amplitude comparison showed that in all but one pair where evoked response was present in both sleep and wakefulness its amplitude was higher in sleep, with no amplitude difference observed in one recording. Thus, although a response can be evoked in wakefulness, it is less likely to occur and when occurs it is normally smaller in amplitude.

This result is indicative of more effective propagation of the visceral signals to cortex during sleep since LFPs stronger represent afferent input (Kappenman and Luck, 2011). To further explore this, we also studied spiking activity of the insular neurons in response to intestinal stimulation in wakefulness and sleep. These results are presented in the next section.

### Neuronal Responses in the Insular Cortex to Electrical Stimulation of Small Intestine

Neuronal activity in sleep and wakefulness was studied in 147 neurons, 61 of them responded to intestinal stimulation (35 cells out of 67 recorded from Cat 1 and 26 out of 80 cells from Cat 2). Dramatic difference was observed in the proportion of insular cells responding to intestinal stimulation in sleep comparing to wakefulness. Cells of the insular cortex were more responsive in sleep. In Cat 1, 24 cells responded to intestinal stimulation exclusively in sleep and only four exclusively in wakefulness. Similarly, in Cat 2, we observed 15 sleep-responsive and seven wakefulness-responsive cells. In addition to that, seven cells of Cat 1 and four of Cat 2 responded in both states. However, in most of these cases neurons changed the sign of their responses—from excitatory responses in one state to inhibitory in the other. In some cases, response latencies were considerably different (>50 ms) in sleep and wakefulness. From 147 neurons studied only one cell in Cat 1 and 2 cells in Cat 2 showed the same responses in sleep and wakefulness.

The above-described breakdown of cell responses to intestinal stimulation by sleep/wakefulness state is summarized in Table 1.

**Table 1:** Numbers of cells responding to intestinal stimulation in sleep and wakefulness.

***N* cells**	**Cat 1**	**Cat 2**
Recorded, total	67	80
Responded, total	35	26
Exclusively SWS-responsive	24	15
Exclusively AWAKE-responsive	4	7
Responded in both conditions, but with opposite signs of the responses	6	2
Responded in both conditions, and with the same response signs	1	2

It is seen that the number of cells exclusively responsive in sleep is significantly bigger in comparison to wakefulness for each of the two cats (*p* < 0.01, Exact Fisher test).

**Figure 6:**
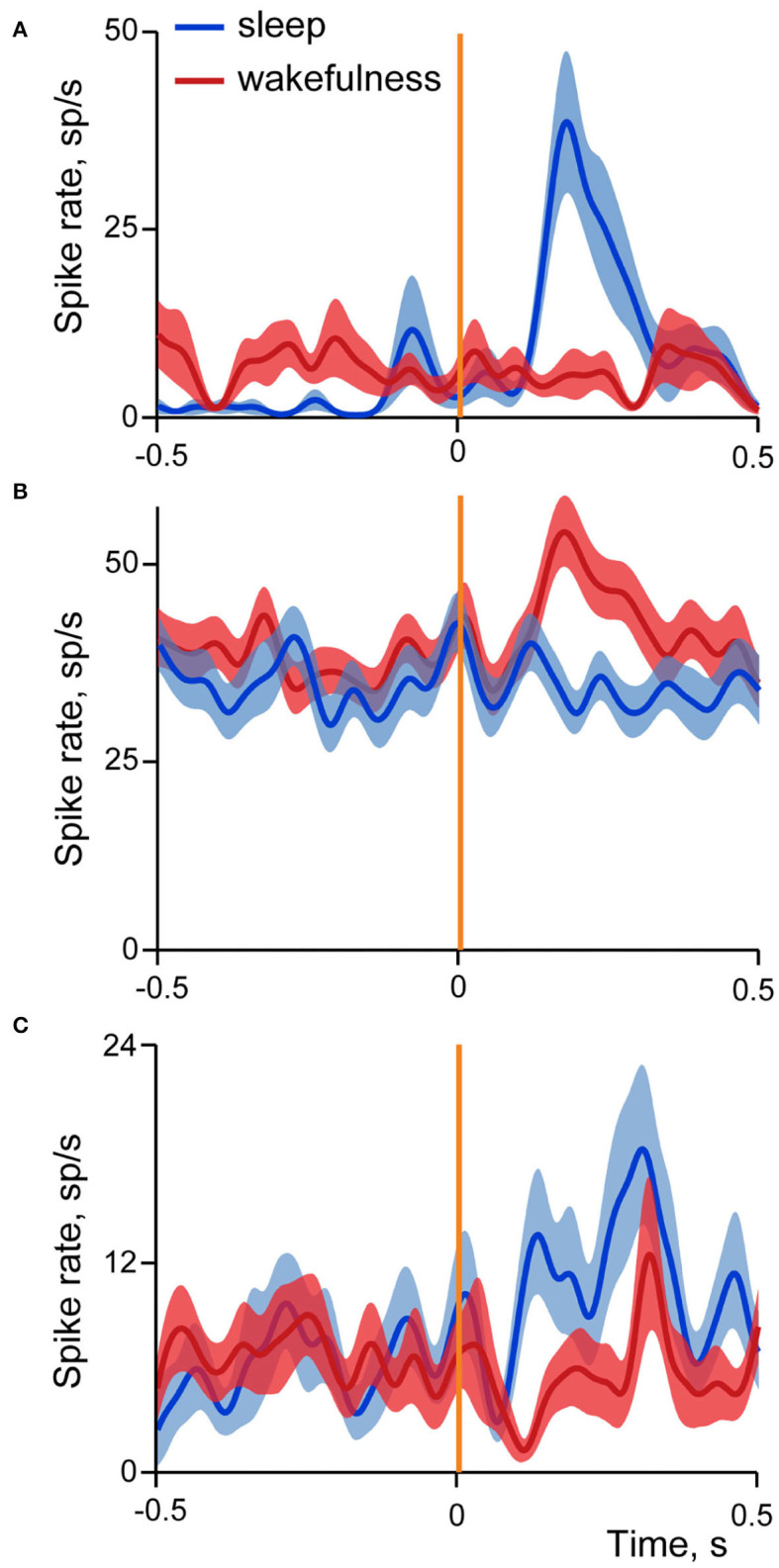
Examples of three types of neuronal responses in the insular cortex evoked by stimulation of small intestine in sleep and wakefulness. Averaged spike rate curves of neurons in sleep are represented by dark blue line, and in wakefulness by dark red line, with standard errors of mean shown in light blue and light red correspondingly. Vertical orange line indicates time of stimulation onset. **(A)** Neuron with excitatory responses in sleep; *N* stimulations in SWS = 18, in wakefulness = 20. **(B)** Neuron with excitatory responses in wakefulness; *N* stimulations in SWS = 51, in wakefulness = 59; **(C)** Neuron with mixed responses, different in sleep and wakefulness; *N* stimulations in SWS = 18, in wakefulness = 21.

Figure 6 shows examples of averaged evoked neuronal responses (spike density curves) of sleep-responding (A), wakefulness-responding (B) and neuron with mixed responses different in sleep and wakefulness (C).

In sleep 79% of cells had excitatory responses, 13% inhibitory and in 8% activity deviated from the baseline in both directions at different time intervals. Similar prevalence of excitatory responses was found in wakefulness (64% excitatory, 27% inhibitory, 9% dual responses).

Since no systematic difference was present between sleep and wakefulness in the number of trials, we conclude that, at the level of neurons, involvement of the insular cortex in processing of information coming from the gastro-intestinal system is more pronounced during sleep. Since most of the insular cells are responsive selectively in either sleep or wakefulness, we conclude that processing of the visceral information by neurons in the insular cortex differs between sleep and wakefulness as well, with the possibility that visceral information reaching cortex in two states can itself be different. Assuming that such processing differences would lead to different executive outcomes and therefore different efferent outflows, we expect that direct electrical stimulation of the insular cortex, applied at the same locations and through the same microelectrodes as the ones used first for recordings of neuronal activity, would lead to different visceromotor and/or behavioral effects depending on the state of vigilance. The next section tests this assumption.

### Behavioral Effects of Insular Electrical Microstimulation Delivered in Wakefulness and Sleep

As previously reported (Olsson and Landgren, 1980; Iwata et al., 1985; Caruana et al., 2011) electrical stimulation of the insular cortex applied in wakefulness evokes contractions of face muscles around a mouth as well as complex orofacial movements.

**Figure 7:**
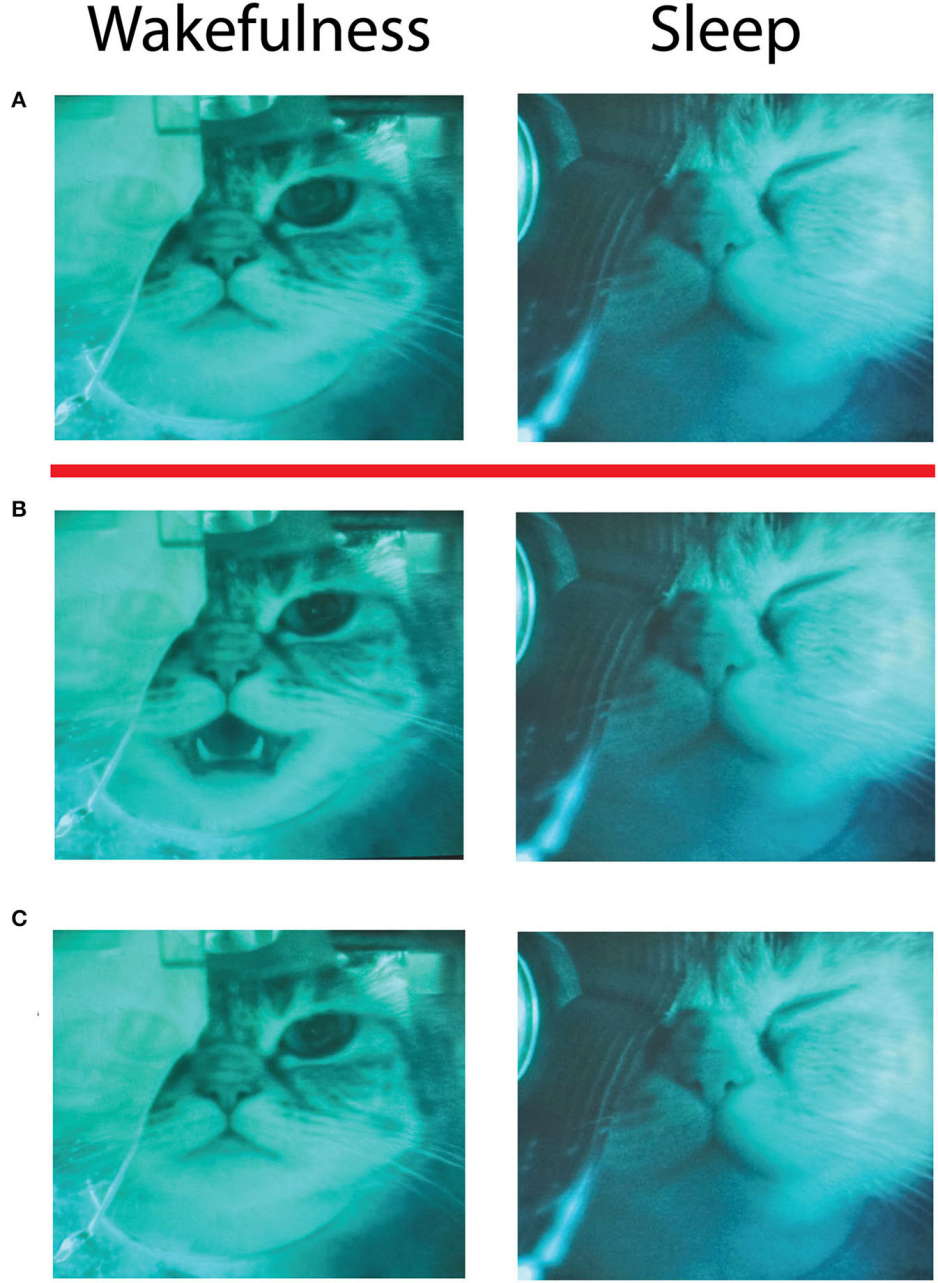
Video frames taken during a session of electrical microstimulation of the insular cortex in wakefulness (left column) and sleep (right column). Red line separates frames taken before the stimulation (**A** above the red line) and after the stimulation (**B,C** below the red line). **(A)** A frame taken immediately before the stimulation, **(B)**−200 ms after the stimulation, **(C)**−3 s after the stimulation. Infrared oculometer can be seen in front of the right eye of the cat. Slight difference of frame brightness between sleep and wakefulness is caused by additional infrared illumination in sleep, when the room lights were dimmed to provide better conditions for sleep.

In our study microstimulations applied during wakefulness in both Cats repeatedly evoked orofacial movements consistent with previous reports (licking, chewing, lip movements, and mouth opening). However, the same stimulating current did not evoke any visible movements of the face muscles during sleep. The motions were diminishing in amplitude when the animals were transitioning from wakefulness to sleep, completely stopping during sleep, and restoring after waking up. The video frames from one such stimulation session are shown in Figure 7. Left column illustrates behavioral effects in wakefulness, and right column—in deep slow wave sleep. The row (A) demonstrates video frames just before electrical stimulation of the insular area. Row (B) shows the frames 200 ms after stimulation, and row (C)—frames taken 3 s after stimulation. It is seen that just after the stimulation in wakefulness a cat opened its mouth, then closed it within 3 s after the stimulation, and did not move after that; the animal continued to sit quietly during and after the stimulation, which indicates that the stimulation was not distressing for the animal. This response to stimulation was well-reproducible, being stereotypical for stimulations applied in wakefulness. With electrode displacement different muscles can be engaged in motion, but in most of the cases contractions were limited to the animal's face. However, in sleep (right column) the same stimulation was absolutely ineffective, and the cat continued to sleep quietly without any observable face movements. This effect of the absence of evoked motions in sleep was robust and present at every recording session. We further used this effect to select the strength of the stimulating current for experiment to avoid disturbing sleep. The current had to evoke clear muscle movements in wakefulness, but not in sleep. These observations indicated that the same electrical stimulation of the insular cortex could indeed produce different effects being delivered in sleep or wakefulness.

### Responses of Duodenal Myoelectrical Activity to Electrical Microstimulation of the Insular Cortex

We performed 25 sessions of insular cortex microstimulation in either right or left hemispheres during recording of intestinal myoelectrical activity in Cat 2. For every stimulation session, stimulations were performed in both sleep and wakefulness for comparability of the evoked effects, and applied to the same insular site within each one recording session. To assess the possible changes of duodenal myoelectric activity, power spectrum density of intestinal activity before and after stimulation were compared in 40 s windows in simple wave frequency range, separately for sleep and wakefulness using Wilcoxon signed rank test. Since changes of simple wave amplitudes are often associated with the occurrence of the spike-potentials, we also analyzed duodenal signal in the spike-potential frequency range, comparing 20 s interval centered at the maximum (or minimum) of the simple wave modulation after the stimulus to the same length interval located at the same distance before the stimulus onset. In sleep seven stimulation sessions led to changes of duodenal simple waves and in five of those sessions to the changes of spike potentials as well, in comparison to the pre-stimulus values.

Stimulations applied in sleep in five sessions led to an increase of the post-stimulus amplitude of the myoelectric signals, and in two sessions amplitude of myoelectrical activity decreased. However, after identical stimulations in wakefulness in the same recording sessions, we did not observe significant response-related changes of intestinal activity.

**Figure 8:**
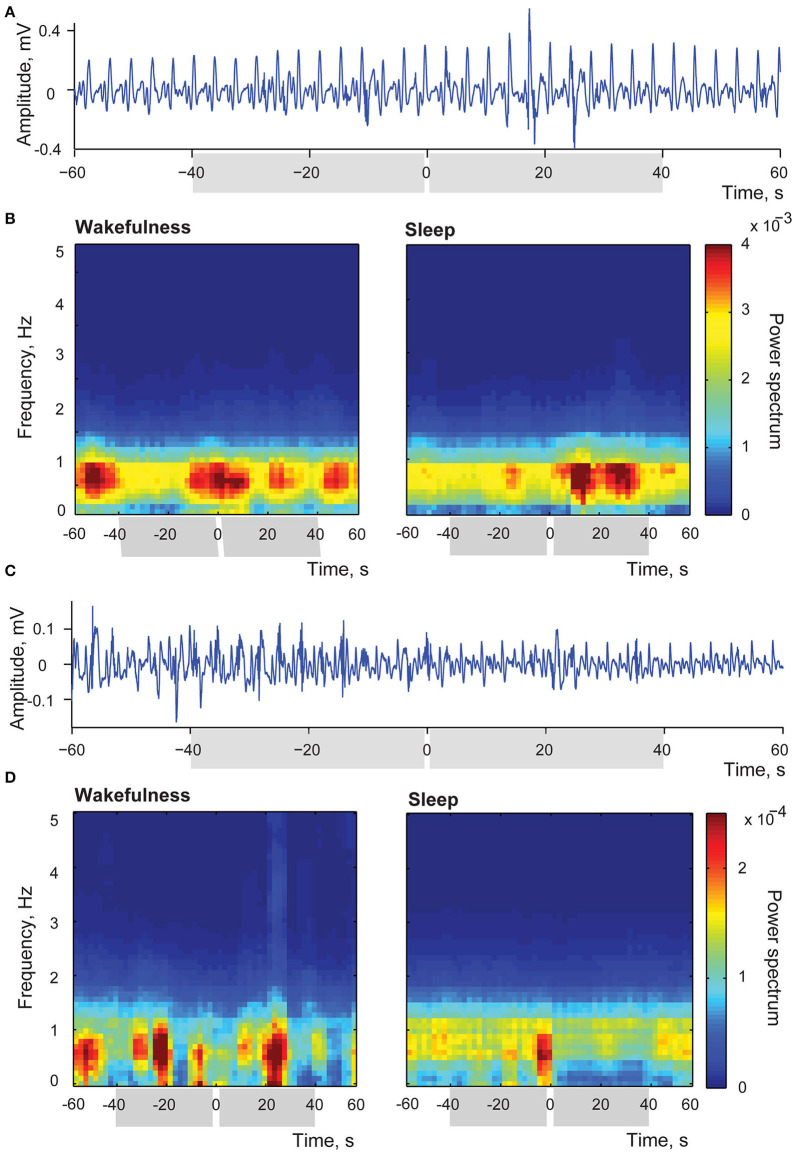
Changes of intestinal myoelectric activity in response to insular microstimulation. **(A)** A single trial example of myoelectric activity amplitude increase after stimulation delivered during slow wave sleep; stimulation occurred at 0 time. **(B)** Averaged time-frequency spectrums of myoelectrical activity recorded from small intestine in the session when stimulations in sleep led to increase of amplitude of this activity while stimulations applied in wakefulness caused no effect. Left panel represents stimulations applied in wakefulness (*N* stimulations = 32), right panel—in sleep (*N* stimulations = 26). Intervals of comparison are indicated by gray rectangles in all panels. **(C)** A single trial example of myoelectric activity decreasing after stimulation delivered during slow wave sleep. **(D)** Averaged time-frequency spectrums of myoelectrical activity recorded from small intestine in the session when stimulations applied in sleep led to decrease of this activity while stimulations applied in wakefulness caused no effect. Left panel represents wakefulness (*N* stimulations = 14), and right panel—sleep (*N* stimulations = 29).

Two examples of the above-mentioned myoelectric activity changes after stimulation of the insular cortex delivered in sleep are shown in Figure 8. Panels (A) and (C) in this Figure provide examples of raw single trial recordings from two different stimulation sessions to illustrate the described changes of the duodenal myoelectrical activity after applied insular stimulation. In the panel (A) amplitude of myoelectric activity increases after the stimulation, and in panel (C) it decreases. Both examples are taking from the recording periods when the animal was in slow wave sleep since no detectable evoked changes were observed in wakefulness.

Panel (B) demonstrates the averaged time-frequency spectrums of myoelectrical activity recorded from the small intestine in the session, when stimulation occurred in sleep led to the entrainment of the myoelectric activity in a way that events of high amplitude activity were aligned, happening more frequently within 40 s after the simulation in comparison to the same length periods before the stimulation (N of averaged stimulations was equal to 26). In the same session in wakefulness (left panel, N of averaged stimulations = 32) activity was not associated with stimulation, with episodes of increased amplitude of duodenal simple waves happening at variable intervals. Such variable amplitude, as previously described for postprandial period (Costa and Furness, 1982), is common to a natural intestinal activity. Thus, in wakefulness duodenal activity was not responsive to the insular stimulation, demonstrating the usual amplitude variation, while in sleep amplitude changes could be evoked by the insular stimulation.

Panel (D) shows the opposite effect of the amplitude decrease in response to stimulation during sleep (29 averaged trials), with the absence of significant difference in wakefulness (14 averaged trials).

To conclude with, motor signals evoked by the stimulation of the insular cortex seem to be effective in producing observable skeletal muscle responses in wakefulness, but not in sleep. Opposite pattern was found in relation to myoelectric intestinal activity, where stimulation led to detectable changes of the intestinal activity only in sleep.

## Discussion

In the introduction to this study we had formulated two alternative hypotheses concerning the general principle of organization of the insular cortex. According to the first, more “classical” view, insular cortex can be the main visceral cortex with constant functions and with permanent afferent and efferent connectivity with the visceral organs. Within this view it would be natural to expect that these properties will be mostly detectable in wakefulness. During sleep thresholds for the visceral inputs would grow, and, as in other cortical areas, neuronal activity would shift toward burst-pause mode of firing and slow waves in EEG. Appearance of these patterns of cortical activity is often associated with recovery processes in the cerebral cortex itself (see e.g., Krueger and Obál, 1993; Kattler et al., 1994; Rector et al., 2005; Huber et al., 2006).

However, during last years another paradigm began to emerge (Pigarev et al., 1997; Pigarev and Pigareva, 2014, 2017). This paradigm was based on unexpected results obtained in investigations of neuronal properties in multiple visual and somatosensory cortical areas. It was found that during sleep neurons in these areas started responding to signals coming from various visceral organs (Pigarev, 1994; Pigarev et al., 2013). These observations indicated that afferent flows to these cortical areas changed to interoceptive in sleep. In such a case burst-pause patterns of neuronal activity during sleep and slow waves in cortical EEG could reflect interference of electrical activity patterns of those visceral afferent flows, which often have rhythmic character (heart beats, respiratory movements, gastro-intestinal peristaltic etc.).

We thought that within this paradigm it would be natural to expect that all cortical areas would demonstrate a single general principle of organization. During sleep, in all cortical areas we expected to observe a replacement of the sensory modality observed in wakefulness by the interoceptive afferentation. Within this approach the view at the insular cortex as the main visceral cortical representation became not so univocal.

How could we combine the available information about the visceral responses of insular neurons in wakefulness with the emerging idea of the change in cortical afferent inflow to the interoceptive one in sleep?

It is widely accepted that the cerebral cortex in wakefulness analyzes signals that carry behaviorally relevant information. These signals we often perceive consciously. We suggest that effective afferent and efferent information flows to and from the insular area in wakefulness are related to those signals from the internal organs that are important for organization of behavior. This can be, e.g., distention of the stomach signaling satiety, or signals from the bladder that requires emptying, etc. These signals reach consciousness, and we can consider them as “special interoceptive.” Indeed, insular cortex is known to be one of the main areas for interoceptive awareness (Craig, 2011).

However, as in the other cortical areas, afferent flow to the insular cortex during sleep changes to the other type of interoceptive afferentation. This afferentation comes from huge number of interoreceptors, which transmit information concerning various parameters of internal organs and body parts, for the purpose of current diagnostic of their state for which cortical preprocessing is crucial. This information under normal circumstances never reaches conscious perception.

Because of such afferentation switch, efferent signaling would also use different pathways, which are exemplified by our results of the insular stimulation: evoked orofacial movements in wakefulness and changes of duodenal activity in sleep.

Thus, we can formulate an alternative hypothesis concerning organization of afferent and efferent connectivity of the insular cortex. In wakefulness insular cortex is engaged in processing of signals that reach consciousness, and which are important for organization of active behavior. However, as in the other cortical areas, connectivity of the insular cortex during sleep changes to processing of interoceptive information that is not supposed to reach consciousness or change behavior, but is likely to affect functioning of the visceral systems. In our opinion results presented in this article correspond to this hypothetical view on the organization of the insular cortex.

### Activity of the Afferent Projections to the Insular Cortex in Sleep—Wake Cycle

Let us analyze first the afferent pathways from duodenum to the insular cortex. As in the previous studies (e.g., Aleksandrov et al., 1996; Bagaev and Aleksandrov, 2006), intestinal stimulation in our experiments indeed provoked responses in the insular cortex in wakefulness supporting conclusion concerning visceral function of this cortical area. These responses were seen in both averaged LFP and in recordings of single units. However, during sleep number of cortical responses to intestinal stimulation was not reduced, but strongly increased, as increased the strength of these responses.

Analysis of neuronal responses to electrical intestinal stimulation revealed another important feature. In most cases, if a neuron responded to stimulation in wakefulness it did not respond to identical stimulation in sleep, and vice versa. There was a relatively small fraction of neurons, which responded in both conditions (eight out of 147). However, their responses were mainly excitatory in one state and inhibitory in another. Only three cells from 147 had similar responses in both states of vigilance (see Table 1).

### Activity of the Efferent Projections From the Insular Cortex in Sleep and Wakefulness

Our study reproduced previous observations (e.g., Olsson and Landgren, 1980; Iwata et al., 1985; Caruana et al., 2011) that electrical stimulation of the insular cortex in wakefulness leads to orofacial movements. However, at the first signs of drowsiness the strength of these movements was reduced and in deep slow wave sleep any movements in response to stimulation disappeared. Video frames shown in Figure 7 exemplify the observed effect. This was not very surprising because skeletal muscle tone normally decreases during sleep. However, deep atonia is usually observed in REM sleep, while we saw the reduction of movements even during transition to sleep, and a lack of observable motions in slow wave sleep. It seems that with the first signs of developing sleep insular cortex quickly attenuated or even stopped communicating with structures involved in organization of behavior (in this case most likely feeding behavior). Since our study did not include direct measurements of a skeletal muscle tone, we cannot provide information regarding the onset of complete atonia of the orofacial muscles; however, it was evident that the same stimulation applied in sleep was unable to produce any visually detectable behavioral changes in comparison to stimulations applied in wakefulness.

For the purpose of this study, it was more important to see whether in sleep signal propagation “opens” from the same points of the insular cortex to some visceral organs, in other words, to observe changes in effective efferentation from the insular cortex to visceral systems. Recording of myoelectrical activity of the duodenum in response to microstimulation of the insular cortex demonstrated that this was indeed the case. In seven out of 25 experiment sessions we observed changes of the myoelectrical activity (simple waves and spike potentials) after stimulation of small areas of the insular cortex using our bipolar microelectrodes capable to activate neurons within a small sphere of about 600−700 μm in diameter. Thus, recording of duodenal myoelectric activity in response to microstimulation of the insular cortex demonstrated changes of the myoelectric activity (simple waves and spike potentials) in sleep.

These observations indicated that the same electrical stimulation of the insular cortex could produce different effects being delivered in sleep or wakefulness. In other words, effective efferent pathways of the insular cortex were different in two states of vigilance.

Ingestion of food is known to create a massive flow of incoming visceral information to the brain, transmitted via vagus nerve but also coming through humoral channels (Furness et al., 2013). Gastrointestinal system sends signals related to distention of its walls as well as to nutrients and other substances coming from the food or secreted internally, in order to optimize digestion, control satiety level and modify food-related behavior. However, it also has the largest epithelial surface area exposed to microbiological and parasitic attacks, therefore existing in a specific state of an ongoing immunological battle, and messages from that front line are also received by the central nervous system. Interestingly, this increase of the amount of transmitted information is also associated with postprandial somnolence—a tendency to sleep after food consumption common to animals or an increased sleep drive and drowsiness experienced by humans after a meal (Antin et al., 1975; Danguir et al., 1979; Orr et al., 1997; Wells et al., 1998) which is likely to reflect partial sleep at the cortical level (Pigarev et al., 1996, 1997; Ferrara and De Gennaro, 2011; Pigarev and Pigareva, 2014).

The soporific effect can be caused by normal increased vagal activity (Leichnetz, 1972; Ramet et al., 1992) and the usual hormonal and nutritional outputs of digestive processes, but also by “visceral error signals,” namely the ones associated with local and generalized inflammation (Juhasz et al., 1985; Bluthé et al., 1996; Simons et al., 1998; Hosoi et al., 2000; Bazar et al., 2004; Burdakov and Alexopoulos, 2005; Burdakov et al., 2005). Thus, specific pattern of increased visceral activity is expected to be transmitted to the brain for processing during complete or partial sleep rather than active wakefulness. It seems reasonable to assume the existence of a specific brain network shaped by visceral information, which would be active predominantly in sleep and specialized for high-order visceral processing.

It is worth mentioning that our research was done using female animals only. At the same time, the insular function is known to vary between genders. The studies showing such effects were conducted mostly in humans as the insula cortex presents a potential therapeutic target for treatment of various diseases of autonomic regulation, which themselves are known to have different gender preponderances. These studies revealed small but significant differences in fMRI signals within the insular lobe, that are associated with sympathetic and parasympathetic regulation of the cardiovascular system (Macey et al., 2016). General difference in insular size with left insula being larger in males corresponding to their bigger brain sizes, and higher gray/white matter ratio in females reflecting their tendency to have higher neuronal density, were also noted (Cosgrove et al., 2007; Rosen et al., 2015). At the same time, using functional MRI, no gender difference was found in the activation of the insular cortex related to food observation (Frank et al., 2010).

Although descriptions of gender differences of the insular functioning in cats are not available, one cannot exclude their existence. However, our study was focused on the general principle of cortical connectivity expressed in wakefulness and sleep which are not likely to differ dramatically between genders.

### Remarks Concerning Probable Non-specific Nature of Cortical Responses to Visceral Stimulation

Demonstration of the cortical responses to visceral stimulation in sleep (Pigarev, 1994; Pigarev et al., 2006, 2013) may cause suspicion that this might not be functional but related to arousing effect of stimulation, or be a representation of other “non-specific” effects such as sensory triggered K-complexes.

In the present study we specially addressed the question of arousing impact of stimulation by examining sleep quality using spectral analysis of EEG and permanent video monitoring before and after application of electrical stimulations. We did not find any indications on arousal in EEG, LFP or behavior. Observed effect of output signal propagation from the insular cortex to small intestine, which occurred only in sleep, further confirmed functional specificity of the cortical visceral processing occurring in sleep.

Regarding sensory provoked “non-specific” K-complexes, our earlier study demonstrated that K-complexes are actually specific effects with mechanisms of generation different from the cortical evoked responses to visceral stimulation in sleep (Pigarev et al., 2011). Sensory provoked K-complexes can be recorded in drowsiness, but can never be seen in deep sleep, while evoked responses to visceral stimulation can occur in deep sleep.

### Possible Pathways for Extero- and Interoceptive Afferentation to the Insular Cortical Areas

Insula is connected to cortical representations of virtually all sensory modalities, as well to associative and motor frontal and parietal areas where multiple visceral inputs were shown anatomically (Öngür and Price, 2000; Saper, 2002; Stephani et al., 2011; Nieuwenhuys, 2012). Moreover, visceral input can also reach these cortical areas via hypothalamus that is known to have bidirectional connections with various prefrontal cortices including insula (Ongür et al., 1998). Retrograde and anterograde tracing experiments involving hypothalamus and periaqueductal gray concluded that areas of so called “medial prefrontal network,” which includes insular cortex, in monkeys project to the lateral, medial, dorsal and posterior hypothalamic zones, involved in visceral regulation (An et al., 1998; Ongür et al., 1998). Multiple cortical areas may receive information from lamina I of spinal pathway, which conveys chemical, metabolic, thermal, and mechanical information from visceral organs (Craig, 2003). These connections can go via the main thalamic nuclei related to visceral signaling: ventral medial nucleus, parafascicular nucleus, and ventral posteromedial nucleus (Beckstead et al., 1980; Pritchard et al., 2000; Ito and Craig, 2005). Another pathway is via the nuclei having input from those spinal pathways where visceral and somatosensory afferents are known to converge to the same neurons (e.g., Cervero et al., 1984; Hobson et al., 2010). In addition, investigation of spinal pathways where visceral and somatosensory information converged revealed that these pathways transmit somatosensory information in wakefulness, while visceral—during sleep (Pigarev et al., 2016).

Lesions or artificial overstimulation of prefrontal and insular cortices produce various disruptions of visceral functioning such as changes in stomach and intestinal motility, vomiting and abdominal pain, hyperglicemia, hypertrophy of the thymus and lymph nodes, hyperplastic bone marrow, arrhythmias, contractile and other cardiac abnormalities and can even lead to sudden cardiac death (Penfield and Faulk, 1955; Neafsey, 1990; Oppenheimer, 1994, 2006; Allport et al., 2004; Tatschl et al., 2006; Nagai et al., 2010; Nieuwenhuys, 2012; Oppenheimer and Cechetto, 2016). On the other hand, several studies demonstrated the role of the insular cortex in organization of complex behavior and cognitive functions (Nieuwenhuys, 2012; Uddin et al., 2014; Billeke et al., 2020). These functions are most definitely associated with active wakefulness.

That points to a possibility for the insular cortex to have a double life—to regulate visceral activity in sleep and to participate in active behavior in wakefulness.

### What Might Be the Role of the Cerebral Cortex in Processing of the Visceral Information?

The question why “some guts” need cerebral cortex to ensure their functioning often arises in discussions of the above described hypothesis.

The reason for this perplexity is most likely the fact that signals coming from interoreceptors located in the visceral organs under normal conditions are not reflected in consciousness. Although the number of interoreceptors is comparable to the number of exteroreceptors, unlike the exterosensation, e.g., visual perception, the processes occurring in the visceral sphere mostly do not reach awareness. Therefore, the complexity of these processes is likely to be underestimated, one can only speculate on the problems that the brain solves during sleep to ensure survival of an organism.

We think that the most general reason might be similar to the one that can be given for the enlargement of cortical parts of other sensory systems in mammals, namely the increase of computational power available for the analysis of complex signals. Diversity of adaptive behaviors including variety of foods consumed, survival despite seasonal changes, and necessity for functional modulations associated with pregnancy, breastfeeding and general prolonged care for offspring probably comes at a price, especially for the species with bigger bodies and longer lifespans. Coordination of visceral functions would require more power due to increased volumes and surface areas of visceral organs and the necessity to support new types of behavior. Adaptation to food variation such as the one demonstrated by monkeys and apes (Passingham and Wise, 2012, p. 57–62) could present new challenges to visceral processing as character of both nutritional intake and microbial invasion would become hugely unpredictable, impacting in turn neuroimmune system, regulation of metabolism and temperature, and indirectly other related visceral functions as well.

During active wakefulness some of the visceral functions might suffer simply due to the necessity to achieve a certain behavioral goal. Activation of sympathetic branch of autonomic regulation is crucial for facing behavioral challenges, but the cost of that is a disruption of some visceral functions, such as the digestive processes, by inhibiting mucosal secretion and motility as well as down regulating blood flow (Costa and Furness, 1982; Browning and Travagli, 2014). Thus, it is hardly surprising that satiety and sleep or some quiet postprandial state are tightly linked in mammals (Antin et al., 1975; Danguir et al., 1979; Orr et al., 1997; Wells et al., 1998) but this association is also noted in other members of animal kingdom including non-vertebrates such as *C. elegans* (Singh et al., 2013). Furthermore, many gastrointestinal maintenance functions are synchronized to circadian cycle, such as prolipheration of gut epithelial cells and gut immune response intensity both peaking at night (Bron and Furness, 2009). Metabolism in general is highly dependent on normal activity of clock system regulating circadian rhythm and on sleep (Woon et al., 2007; Takahashi et al., 2008; Copinschi et al., 2014), and sleep deprivation is known to wreak havoc in visceral functioning, with complete deprivation for prolonged period leading to inevitable death (Rechtschaffen and Bergmann, 2002; Periasamy et al., 2015). The link between sleep and certain types of visceral signaling such as the ones described above probably creates developmental drive leading to formation of the cortical network active in sleep.

Discovered change of the afferent flow to the same cortical areas indicates that these areas are not specialized processors for analysis of only one particular type of sensory modality, but are universal pre-processors, which perform some standard operations with incoming information independent of its meaning. After this preliminary, but complicated, processing, resulting information should be directed in wakefulness to those brain regions, which are involved in organization of behavior and motor activity (most likely to structures of basal ganglia). During sleep cortical outflows should be directed toward the brain structures providing high level of associative visceral control (e.g., to hypothalamus). This is consistent with described positive changes of glucose consumption in the hypothalamus during sleep, in contrast to negative changes happening in the structures heavily involved in wakefulness-dependent exteroceptive functions, such as basal ganglia and thalamus (Ramm and Frost, 1983, 1986; Buchsbaum et al., 1989; Nofzinger et al., 2002).

Optimal solution for life on the planet with periodical day-night shift probably is “sharing” of computational resources—sequential use of the same big and powerful brain part-time for organization of active behavior and part-time for diagnostic and functional recovery of all life supporting systems and organs.

## Conclusion

Our results demonstrate the preponderance of the insular cortex to the analysis of visceral information in sleep in comparison to wakefulness. We would like to attract attention to the cortical involvement in visceral regulation, which is manifested during periods of sleep. These periods of sleep are informative not only for comprehension of the nature of cortical organization and activity, but are also clinically important for understanding the relationships between visceral issues, sleep, and cortical abnormalities.

## Data Availability Statement

The raw data supporting the conclusions of this article will be made available by the authors, upon the reasonable request.

## Ethics Statement

Ethical review and approval was not required for the animal study because Surgery and treatment of the animals were carried out in accordance with the Ethical Principles for the maintenance and use of animals in neuroscience research (Zimmermann, 1987), NIH guidelines for the care and use of animals and Declaration of Helsinki on Ethical Principles for Medical Research. Current Russian laws do not bind scientific Institutes to have special Ethic committees; instead, assessment of the ethical components of a research proposal is conducted by the Institutional Scientific Councils. According to the practice of the Russian Foundations distributing funding for scientific grants, the Council of Reviewers prior to making a decision regarding financial support of a particular study performs ethics evaluation. Both Councils are guided by the recommendations of the above-mentioned documents.

## Author Contributions

EL and IP contributed to conception of the idea and design of the study, collected the data, and wrote the first draft of the manuscript. IB, MP, and IP performed sergeries. All authors contributed to data analysis and manuscript revision, read and approved the submitted version of the paper.

## Conflict of Interest

The authors declare that the research was conducted in the absence of any commercial or financial relationships that could be construed as a potential conflict of interest.
